# Protective Effect of 2,4′,5′-Trihydroxyl-5,2′-dibromo diphenylmethanone, a New Halophenol, against Hydrogen Peroxide-Induced EA.hy926 Cells Injury

**DOI:** 10.3390/molecules200814254

**Published:** 2015-08-05

**Authors:** Jianguo Li, Xiue Feng, Rui Ge, Jiankuan Li, Qingshan Li

**Affiliations:** 1 School of Pharmaceutical Science, Shanxi Medical University, 56 Xinjian South Road, Taiyuan 030001, China; E-Mails: ljg2547@163.com (J.L.); xiuefeng@163.com (X.F.); cpugr@126.com (R.G.); lijiankuan2004@163.com (J.L.); 2 School of Public Health Science, Shanxi Medical University, Taiyuan 030001, China; 3 Shanxi Key Laboratory of Drug Toxicology and Drug for Radiation Injury, China Institute for Radiation Protection, Taiyuan 030006, China

**Keywords:** 2,4′,5′-trihydroxyl-5,2′-dibromo diphenylmethanone, EA.hy926 cells, H_2_O_2_, apoptosis, microarray analysis

## Abstract

Vascular endothelial cells produce reactive oxygen species (ROS) during the process of energy metabolism in aerobic respiration. A growing body of evidence indicates that excessive ROS is implicated in the pathogenesis of cardiovascular diseases including atherosclerosis. The newly synthesized halophenol, 2,4′,5′-trihydroxyl-5,2′-dibromo diphenylmethanone (TDD), exhibits antioxidative and cytoprotective activities *in vitro*. In this study, the protective effect of TDD against hydrogen peroxide (H_2_O_2_)-induced oxidative injury of EA.hy926 cells was investigated. Cell viability was measured by 3-(4,5-dimethylthiazol-2-yl)-2,5-dephenyltetrazolium bromide (MTT) assay, while the effect of TDD on the transcription profile of EA.hy926 cells subjected to H_2_O_2_-induced oxidative injury was evaluated by microarray analysis. Several signaling pathways, including apoptosis, were significantly associated with TDD. Flow cytometric analysis was used to evaluate anti-apoptotic effect of TDD. Subsequently, RT-PCR and Western blot were used to detect the expressions of the apoptosis-associated protein, Bcl-2 and Bax. Meanwhile the expression of cleaved caspase-3, an executioner of apoptosis, was also detected by Western blot. The results showed that pretreatment of EA.hy926 cells with TDD prevented the decrease of cell viability induced by H_2_O_2_, and attenuated H_2_O_2_-induced elevation of Bax and cleaved caspase-3 while increased Bcl-2 expressions. In summary, TDD inhibited H_2_O_2_-induced oxidative injury of EA.hy926 cells through negative regulation of apoptosis. These findings suggest that TDD is a potential candidate for therapeutic intervention in oxidative stress-associated cardiovascular diseases.

## 1. Introduction

Reactive oxygen species (ROS), including superoxide anion (O_2_^−^), hydroxyl radical (HO^−^), and hydrogen peroxide (H_2_O_2_), are produced during the process of energy metabolism as part of aerobic respiration in cells. ROS plays an important role in the induction of cell apoptosis under both physiologic and pathologic conditions [1]. Apoptosis, also known as programmed cell death, is a process in which potentially dangerous and unwanted cells are eliminated from tissues to maintain organism homeostasis. Dysregulation of apoptosis leads to a variety of human diseases, such as cancer, autoimmune diseases, and neurodegenerative disorders [2]. Increasing evidence suggests that excessive levels of ROS in the vascular endothelium results in damaged endothelial NO bioactivity, abnormally up-regulated expression of cell surface adhesion molecules, and increased inflammatory changes, which contribute to the vascular pathogenesis of atherosclerosis, hypertension, and heart failure [3,4,5,6,7]. Apoptosis of endothelial cells may destroy vascular barrier integrity and result in increased endothelial permeability, platelet aggregation, leukocyte adhesion, and generation of cytokines [8]. Hence, endothelial cell apoptosis is considered to be partially responsible for the pathogenesis of cardiovascular diseases, such as atherosclerosis, hypertension, thrombosis, and heart failure [9]. These findings have promoted the search for new pharmacological approaches to decrease and/or prevent oxidative stress-induced vascular damages.

**Figure 1:**
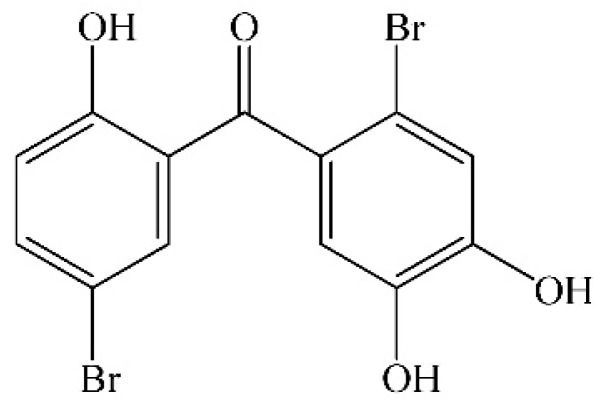
Chemical structure of 2,4′,5′-trihydroxyl-5,2′-dibromo diphenylmethanone.

Halophenols, isolated from halobios such as various marine algae, ascidians and sponges exhibit a wide spectrum of bioactivities including protein tyrosine phosphatase (PTP1B) inhibitory [10], antioxidative [11,12], antithrombotic [13], antimicrobial [14,15], anti-inflammatory [16], enzyme inhibitory [17], cytotoxic [18], appetite suppressant [19], and PTK inhibitory activities [20]. Recently, we synthesized a variety of halophenols including 2,4′,5′-trihydroxyl-5,2′-dibromo diphenylmethanone (TDD, Figure 1), which has strong antioxidative and cytoprotective activities [21,22,23]. However, the detailed mechanisms underscoring its actions are unclear. In this paper, we investigated the protective effect of TDD on oxidative stress-induced injury of EA.hy926 cells.

## 2. Results and Discussion

### 2.1. Cell Viability

**Figure 2:**
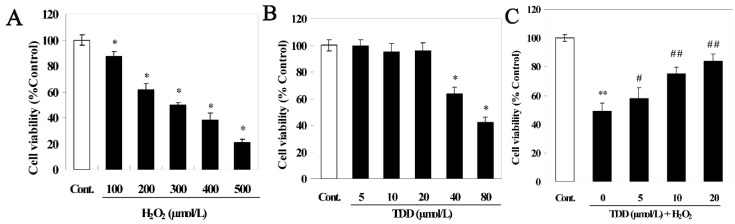
Protective activity of 2,4′,5′-trihydroxyl-5,2′-dibromo diphenylmethanone was measured by the MTT assay. (**A**) Cell viability of EA.hy926 cells treated with different concentration of H_2_O_2_; (**B**) Cell viability of EA.hy926 cells treated with different concentration of TDD; (**C**) Cell viability of EA.hy926 cells treated with TDD followed by H_2_O_2_ treatment. Data are presented as mean ± SD (*n* = 6). * *p* < 0.05 and ** *p* < 0.01 *vs.* control; **^#^** *p* < 0.05 and **^##^** *p* < 0.01 *vs.* H_2_O_2_ group.

The MTT assay showed that H_2_O_2_ decreased cell viability in a concentration-dependent manner. As shown in Figure 2A, the viability of cells exposed to H_2_O_2_ at 100, 200, 300, 400, and 500 μM for 24 h decreased to 87.53% ± 3.57%, 61.73% ± 4.55%, 49.75% ± 2.03%, 38.42% ± 4.96%, and 20.62% ± 2.53%, respectively, of the non-treated control value (normalized to 100%). Because exposure to 200 μM H_2_O_2_ for 24 h reduced cell survival in a modest but readily detectable manner, this dose was used to induce EA.hy926 cell injury in subsequent experiments. The cytotoxicity of TDD alone was also evaluated by MTT assay. As shown in Figure 2B, treatment with less than 20 μM TDD for 24 h had no significant effect on the viability of EA.hy926 cells. Next, we investigated whether TDD provided protection against H_2_O_2_-induced cytotoxicity on EA.hy926 cells. To do this the EA.hy926 cells were pretreated with 5, 10, or 20 μM TDD followed by exposure to 200 μM H_2_O_2_, and then cell viability was detected with the MTT assay. As shown in Figure 2C, pretreatment of cells with TDD attenuated H_2_O_2_-induced loss-of-viability in a concentration-dependent manner, suggesting that TDD may protect cells from oxidative stress-induced cytotoxicity.

### 2.2. Microarray Analysis

To further examine the effect of TDD on H_2_O_2_-induced inhibition of cell survival, microarray analysis was performed whereby data fluctuations above 2-fold were considered to represent significant differential expression. When compared with data from H_2_O_2_-treated cells and TDD-pretreated cells followed by H_2_O_2_ treatment resulted in the significantly changed expression of 1083 genes. Of these, 419 were up-regulated, while 664 were down-regulated.

Gene Ontology (GO) analysis [24] of microarray data was performed to obtain an overview of the cellular physiologic status of H_2_O_2_-exposed EA.hy926 cells pretreated with TDD. The GO database is organized into three categories including biological process, molecular function, and cellular component. GO terms were considered significant if they contained at least 9 genes with *p* values below 1.0 × 10^−2^. According to GO analysis, biological processes influenced by either H_2_O_2_ alone or in combination with TDD were mainly cellular nitrogen compound metabolism, negative regulation of apoptosis, regulation of small GTPase mediated signal transduction, cytokine-mediated signaling, aging, RNA processing, and response to lipopolysaccharide (Table 1), which demonstrated that TDD participated in the regulation of apoptosis, inflammation, and other basic process of cell life activities in H_2_O_2_-induced EA.hy926 cells.

**Table 1:** Major biology processes.

Biological Process	Gene Number	*p* Value
Cellular nitrogen compound metabolic process	14	6.95 × 10^−3^
Negative regulation of apoptosis	13	1.33 × 10^−2^
Regulation of small GTPase mediated signal transduction	13	5.42 × 10^−3^
Cytokine-mediated signaling pathway	10	4.51 × 10^−2^
aging	10	3.17 × 10^−3^
RNA processing	9	2.08 × 10^−3^
Reponse to lipopoly saccharide	9	2.2 × 10^−2^

**Figure 3:**
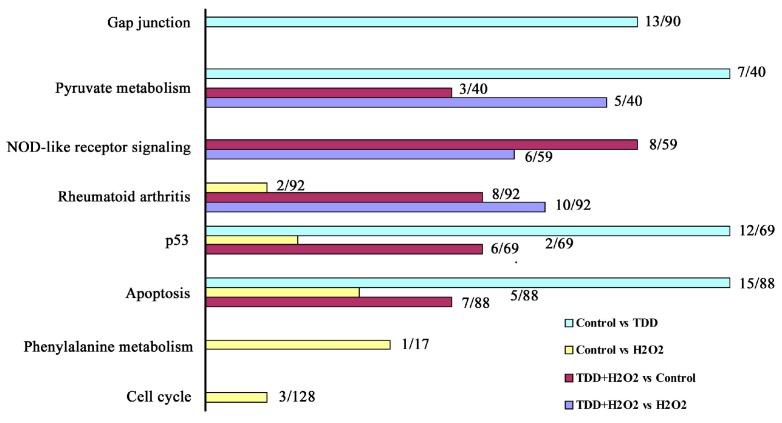
Pathways closely related with differentially expressed genes in different groups.

The biological pathways that were significantly (*p* < 0.05) affected by the differential gene expression were also investigated. Figure 3 shows the results of pathway analysis of the differentially expressed genes among different groups. The results showed that apoptosis and p53 were common to three comparisons between control *vs.* TDD, control *vs.* H_2_O_2_, and control *vs.* TDD + H_2_O_2_, which indicated that TDD was likely involved in apoptosis. As a tumor suppressor gene, p53 was an apoptosis-associated gene to induce apoptosis of cancer cells. Thus, we proposed the hypothesis that the protective effect of TDD against H_2_O_2_-induced EA.hy926 injury maybe related with apoptotic pathway, which was proved by analysis of apoptosis-associated gene/protein with RT-PCR and Western blot in following experiments. Meanwhile, other biological pathways including rheumatoid arthritis, NOD-like receptor, pyruvate and phenylalanine metabolism, cell cycle and gap junction were exhibited in different comparisons. NOD-like receptor pathway played a key role in the host innate immune system and was associated with the pathology of several inflammatory diseases including rheumatoid arthritis [25,26]. Pyruvate and phenylalanine metabolism, and cell cycle were basic process of cell life activities. Gap junction played key role in the establishment of communication between endothelial cell to cell [27]. These results demonstrated that TDD may also affect these pathways except for apoptosis, which need to be clear.

### 2.3. Flow Cytometic Analysis of Apoptosis

**Figure 4:**
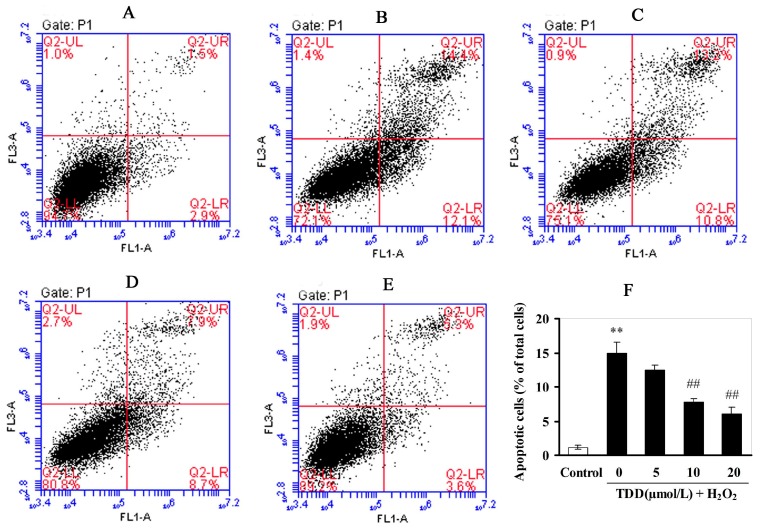
Effects of 2,4′,5′-trihydroxyl-5,2′-dibromo diphenylmethanone on H_2_O_2_-induced apoptosis in EA.hy926 cells measured by flow cytometry. (**A**) Control group; (**B**) H_2_O_2_ treatment group; (**C**–**E**) 5, 10, and 20 μmol/L TDD, respectively, followed by the treatment of 200 μmol/L H_2_O_2_; (**F**) TDD decreased the percent of apoptotic cells induced by 200 μmol/L H_2_O_2_ in a dose-dependant manner. Data are presented as the mean ± SD (*n* = 3); ** *p* < 0.05 compared with the control; **^##^** *p* < 0.01 compared with the H_2_O_2_-treated cells.

The protective effect of TDD against H_2_O_2_-induced apoptosis in EA.hy926 cells was evaluated by flow cytometic analysis. As shown in Figure 4, the percentage of apoptotic cells (upper right) was increased from 1.2% in the control group to 14.9% in the H_2_O_2_ only treated group. However, pretreatments of the cells with 5, 10, and 20 μmol/L TDD significantly decreased the cell apoptotic rates induced by H_2_O_2_ to 12.5%, 7.7%, and 6.1%, respectively. The results suggested that TDD protected EA.hy926 cells against H_2_O_2_-induced apoptosis.

### 2.4. Quantitative RT-PCR Analysis

**Figure 5:**
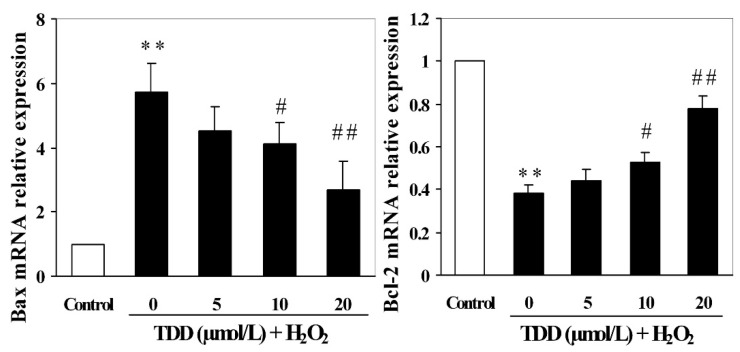
Effects of 2,4′,5′-trihydroxyl-5,2′-dibromo diphenylmethanone on the expression of Bcl-2 mRNA and Bax mRNA in H_2_O_2_-induced EA.hy926 cells by real-time PCR. Data are presented as mean ± SD (*n* = 3). ** *p* < 0.01 *vs.* control; **^#^** *p* < 0.05 and **^##^** *p* < 0.01 *vs.* H_2_O_2_ group.

To determine whether the protective effect of TDD against H_2_O_2_-induced apoptosis in EA.hy926 cells was related to the modulation of apoptosis-associated gene expression, the mRNA transcription levels of Bcl-2 and Bax were detected by RT-PCR. As shown in Figure 5, treatment of EA.hy926 cells with 200 μmol/L H_2_O_2_ significantly increased Bax mRNA expression, while it significantly decreased that of Bcl-2 compared with the control group (*p* < 0.01). However, pretreatment with TDD at concentrations of 5, 10, and 20 μmol/L remarkably enhanced Bcl-2 mRNA expression and inhibited Bax mRNA expression in a dose-dependent manner.

### 2.5. Western Blot Analysis

**Figure 6:**
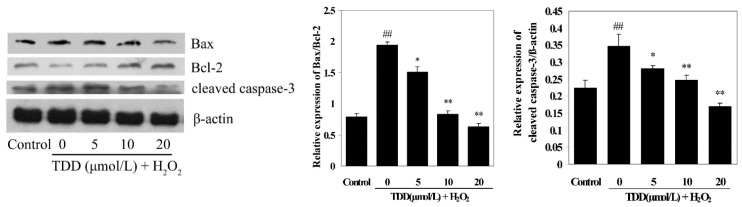
Effects of 2,4′,5′-trihydroxyl-5,2′-dibromo diphenylmethanone on the expression of Bcl-2, Bax and cleaved caspase-3 in H_2_O_2_-induced EA.hy926 cells by Western blot. Data are presented as mean ± SD (*n* = 3). ** *p* < 0.01 *vs.* control; * *p* < 0.05 and **^##^** *p* < 0.01 *vs.* H_2_O_2_ group.

Western blot was used to detect the expressions of apoptosis-associated proteins Bax, Bcl-2 and cleaved caspase-3. As shown in Figure 6, compared with the control group, treatment of EA.hy926 cells with 200 μmol/L H_2_O_2_ significantly increased Bax and cleaved caspase-3 expression, while it significantly decreased Bcl-2 expression (*p* < 0.01). However, pretreatment with TDD at concentrations of 5, 10, and 20 μmol/L obviously enhanced Bcl-2 expression and inhibited Bax and cleaved caspase-3 expression in a dose-dependent manner.

The Bcl-2 (B-cell lymphoma-2) family includes important proteins that are involved in the mitochondrial apoptosis pathway. Among them, Bcl-2 is an antiapoptotic protein, whereas Bax is a proapoptotic protein. Indeed, proapoptotic Bax is reported to control mitochondrial permeability and the subsequent release of cytochrome *c*, which leads to the activation of caspase-3 and apoptosis, while antiapoptotic Bcl-2 inhibits Bax [28]. According to GO analysis, apoptosis was a major process involved in EA.hy926 cell injury induced by either H_2_O_2_- alone or in combination with TDD. Therefore we measured the mRNA and protein expression of Bcl-2 and Bax by RT-PCR and Western blot respectively. We showed that TDD significantly prevented both H_2_O_2_-induced decrease of Bcl-2 mRNA and protein expression and increase of Bax mRNA and protein expression levels, indicating that the protective effects of TDD against H_2_O_2_-induced apoptosis may be associated with the modulation of the Bax and Bcl-2.

Caspase components play a central role in the execution of apoptosis [29,30]. There are two main well characterized caspase-activating pathways that modulate apoptosis: Death receptor pathway (extrinsic) and mitochondrial pathway (intrinsic). Caspase-3, an executioner of apoptosis for both extrinsic and intrinsic pathways, is cleaved and activated in the process of apoptosis. The current study demonstrated that caspase-3 was cleaved after 24 h of 200 μM H_2_O_2_ treatment in EA.hy926 cells. Pretreatment of the cells with TDD attenuated H_2_O_2_-induced expression of cleaved caspase-3 in a dose-dependent manner, strongly supporting the protective effect of TDD against H_2_O_2_-induced apoptosis in EA.hy926 cells.

## 3. Experimental Section

### 3.1. Chemicals

2,4′,5′-Trihydroxyl-5,2′-dibromo diphenylmethanone (TDD) was synthesized by our research group. Its purity was determined to be 99% by HPLC analysis. Dimethyl sulfoxide (DMSO), hydrogen peroxide, 3-(4,5-Dimethylthiazol-2-yl)-2,5-Dephenyltetrazolium bromide (MTT), and 4% paraformaldehyde were obtained from Sigma Aldrich (St. Louis, MO, USA). Dulbecco’s modified Eagle’s medium (DMEM), l-glutamine, penicillin, and streptomycin were purchased from Gibco BRL (Grand Island, NY, USA). Annexin V-FITC apoptosis detection kit was purchased from Nanjing KeyGen Biotech. Co., Ltd. (Nanjing, China). All other chemicals used were of analytical grade. H_2_O_2_ was freshly prepared for each experiment from a 33% stock solution.

### 3.2. Cell Culture and Drug Treatments

The EA.hy926 human vascular endothelial cell line was purchased from the Cell Bank of the Chinese Academy of Sciences (Shanghai, China) and maintained in high-glucose DMEM supplemented with 10% fetal bovine serum (FBS; Hyclone, Beijing, China), l-glutamine (2 mM), 100 units/mL penicillin, and 100 mg/mL streptomycin. Cells were incubated in a humidified incubator aerated with 5% CO_2_ at 37 °C. For all experiments, EA.hy926 cells were grown to 70%–80% confluence and then pretreated with the designated concentrations of TDD for 60 min prior to H_2_O_2_ exposure in fresh medium.

### 3.3. Cell Viability Assay

The MTT assay was used to evaluate cell viability. EA.hy926 cells (1 × 10^4^ per well) were seeded in 96-well plates and cultured for 24 h, then the medium was replaced with fresh medium for different treatments. After treatments, 10 μL 5 mg/mL MTT, diluted in phosphate buffered saline (PBS), was added to each well and cells were further incubated for 4 h. Then, culture medium was removed and 100 μL DMSO was added per well to dissolve any formed precipitate. The absorbance of samples was measured at a wavelength of 490 nm on a Bio-Rad Microplate Reader (Model 680; Bio-Rad, San Diego, CA, USA).

### 3.4. Preparation of Reference RNA

EA.hy926 cells were seeded at 6 × 10^4^ cells in 25 cm^2^ cell culture flasks and incubated for 24 h. Culture medium was removed and the cells were incubated with 5 mL complete medium containing 10 μmol/L TDD for 1 h, then exposed to 200 μmol/L H_2_O_2_ for 24 h. After these treatments, total RNA was isolated using the mirVana™ RNA Isolation Kit (Applied Biosystems, Austin, TX, USA) according to the manufacturer’s procedure. RNA purity and concentration were determined by the Agilent 2100 bioanalyzer (Agilent, Santa Clara, CA, USA). RNA samples were stored at −80 °C until further analysis.

### 3.5. Microarray Analysis

Gene expression analysis of the RNA samples was performed using the Agilent Human 8 × 60 K. The microarray assay was carried out by OEBIOTECH (Shanghai, China).

### 3.6. Flow Cytometic Analysis of Apoptosis

Annexin V-FITC/propidium iodide (PI) double-staining kit (Life Technologies, Eugene, OR, USA) was used to detect apoptosis. After treatment with various concentrations of TDD or/and H_2_O_2_, EA.hy926 cells were collected and washed with ice-cold PBS twice, and then resuspended in binding buffer at a concentration of 1 × 10^6^ cells/mL. Then, 5 μL of annexin V-FITC and 5 μL of PI were added. The cells were incubated for 15 min in the dark and then the quantification of apoptosis was analyzed using flow cytometry (Becton Dickinson, Franklin Lakes, NJ, USA).

### 3.7. RNA Reverse Transcription and Quantitative RT-PCR

Total RNA (1 mg) from each test group used for the RT-PCR. It was first reverse-transcribed to cDNA using the Revert Aid first strand cDNA synthesis kit (Sangon Biotech; Shanghai, China) and stored at −20 °C. In accordance with the microarray data, Bcl-2 and Bax were selected for further analysis by RT-PCR. Primers specific to the genes of interest, as well as those for the internal control GAPDH were used for amplification; their forward and reverse sequences were as follows: Bcl-2,5′-atgtgtgtggagagcgtcaac-3′ and 5′-agagacagccaggagaaatcaaac-3′; Bax, 5′-aagctgagcgagtgtctcaag-3′ and 5′-caaagtagaaaagggcgacaac-3′; GAPDH, 5′-tgaacgggaagctcactgg-3′ and 5′-tccaccaccctgttgctgta-3′. Following initial denaturation at 95 °C for 15 min, the amplification conditions were 40 cycles of denaturation at 95 °C for 10 s, annealing at 60 °C for 30 s, and elongation at 72 °C for 20 s. All PCR data were checked for homogeneity by dissociation curve analysis.

### 3.8. Western Blot Analysis

Western blot was performed as reported by our colleagues [24]. Briefly, after treatment with various concentrations of TDD or H_2_O_2_, EA.hy926 cells were washed with ice-cold PBS and harvested with 0.25% trypsin. Then the cells were centrifuged at 1000 rpm for 5 min and washed with ice cold PBS for 3 times. Then the cells were suspended in 100 μL ice-cold RIPA lysis buffer and sonicated 10 times for 5 s with 10 s pauses in an ice-water bath and centrifuged at 10,000 rpm for 10 min at 4 °C. Protein quantity was detected with BCA assay kit, and equal amount of proteins were used for Western blot analysis.

For Western blot analysis, equal amounts of protein extracts (50 μg) were separated by 12% SDS-polyacrylamide gels, and then proteins were transferred onto nitrocellulose (NC) membrane (PALL Gelman Laboratory, New York, NY, USA). The membrane was blocked in 5% nonfat milk powder in Tris-buffered saline/0.1% Tween-20 (TBST) for 1.5 h at room temperature, and then incubated overnight at 4 °C with the primary antibodies. After three washes with TBST, the membrane was incubated for 1 h with horseradish peroxidase (HRP)-conjugated anti-rabbit IgG as the secondary antibody at room temperature. After three times washes, the immune blots were detected by enhanced chemiluminescence (ECL) detection kit (CoWin Biotech Co., Ltd., Beijing, China). The quantification of bands was performed according to densitometry using Adobe Photoshop software (7.0.1, Adobe, San Jose, CA, USA).

### 3.9. Statistical Analysis

Each experiment was performed at least three times. The values were expressed as the mean ± standard deviation (SD). Differences among experimental groups were evaluated by Student’s *t*-test. Values of *p* < 0.05 were considered statistically significant.

## 4. Conclusions

In conclusion, our findings provide evidence that TDD exerts a protective effect against H_2_O_2_-induced apoptosis in EA.hy926 cells which is likely mediated through regulation of Bax and Bcl-2 expression. These data suggest that TDD is a potential candidate for intervention to reduce and/or prevent oxidative stress in cardiovascular diseases including atherosclerosis.
